# α-Lys^424^ Participates in Insertion of FeMoco to MoFe Protein and Maintains Nitrogenase Activity in *Klebsiella oxytoca* M5al

**DOI:** 10.3389/fmicb.2019.00802

**Published:** 2019-04-16

**Authors:** Lina Song, Pengxi Liu, Wei Jiang, Qingjuan Guo, Chunxi Zhang, Abdul Basit, Ying Li, Jilun Li

**Affiliations:** ^1^State Key Laboratory of Agrobiotechnology, College of Biological Sciences, China Agricultural University, Beijing, China; ^2^Laboratory of Photochemistry, Institute of Chemistry, Chinese Academy of Sciences, Beijing, China

**Keywords:** *Klebsiella oxytoca*, nitrogenase, MoFe protein, α-Lys^424^, FeMoco

## Abstract

Our previous investigation of substrates reduction catalyzed by nitrogenase suggested that α-Ile^423^ of MoFe protein possibly functions as an electron transfer gate to Mo site of active center-“FeMoco”. Amino acid residue α-Lys^424^ connects directly to α-Ile^423^, and they are located in the same α-helix (α423-431). In the present study, function of α-Lys^424^ was investigated by replacing it with Arg (alkaline, like Lys), Gln (neutral), Glu (acidic), and Ala (neutral) through site-directed mutagenesis and homologous recombination. The mutants were, respectively, termed 424R, 424Q, 424E, and 424A. Studies of diazotrophic cell growth, cytological, and enzymatic properties indicated that none of the substitutions altered the secondary structure of MoFe protein, or normal expression of *nifA*, *nifL*, and *nifD*. Substitution of alkaline amino acid (i.e., 424R) maintained acetylene (C_2_H_2_) and proton (H^+^) reduction activities at normal levels similar to that of wild-type (WT), because its FeMoco content did not reduce. In contrast, substitution of acidic or neutral amino acid (i.e., 424Q, 424E, 424A) impaired the catalytic activity of nitrogenase to varying degrees. Combination of MoFe protein structural simulation and the results of a series of experiments, the function of α-Lys^424^ in ensuring insertion of FeMoco to MoFe protein was further confirmed, and the contribution of α-Lys^424^ in maintaining low potential of the microenvironment causing efficient catalytic activity of nitrogenase was demonstrated.

## Introduction

Nitrogen can be utilized only in combined forms, created by “nitrogen fixation”. Biological nitrogen fixation depends on nitrogenase (Davis, 2002). Nitrogenase reduces dinitrogen (N_2_) to ammonia (NH_3_) at normal ambient temperature and pressure (Rees, 1993; Milton et al., 2017). Various types of nitrogenase are present in different microorganisms (Bishop et al., 1980; Eady et al., 1988; Joerger et al., 1990). Mo-dependent nitrogenase is the most widely studied and composed by Fe protein (NifH; ∼70 kD) and MoFe protein (NifDK; ∼240 kD) (Bulen and Lecomte, 1966; Burgess and Lowe, 1996; Einsle et al., 2002). Fe protein is a γ_2_ dimer containing a [4Fe-4S] cluster, the original electron acceptor of nitrogenase (Georgiadis et al., 1992; Kim and Rees, 1992). MoFe protein is an α_2_β_2_ tetramer and composed of two metal clusters: P-cluster ([8Fe-7S] cluster) and FeMoco (“FeMo-cofactor”; 7Fe-9S-C-Mo-homocitrate) (Gemoets et al., 1989; Jongsun and Rees, 1992; Mayer et al., 1999). FeMoco is the active center of nitrogenase, and is the most sophisticated metal cluster in the organisms. Its structure was not fully elucidated until 2011, when the light atom in the central cavity was confirmed to be a C-atom by several methods (Lancaster et al., 2011; Spatzal et al., 2011). FeMoco is anchored to MoFe protein via the covalent bonding with α-Cys^273^ and α-His^440^, and is surrounded by a series of residues (Kim et al., 1995; Igarashi and Seefeldt, 2004; Yang et al., 2011). Homocitrate is positioned at the Mo terminal of FeMoco and plays a key role in nitrogenase activity (Mayer et al., 2002).

Major research progress has been made, particularly in regard to nitrogenase structure and the reduction of non-biological substrates (Lancaster et al., 2011; Sippel and Einsle, 2017). However, several major points remain unclear. Nitrogen reduction requires MgATP supplementation to Fe protein during every catalytic cycle (Danyal et al., 2011), but it is unclear how many MgATPs are required to pass one electron. The nitrogen binding site on FeMoco remians controversial because of the difficulty of tracking the route for binding (De et al., 2017; Sickerman et al., 2017). A series of Mo-dependent compounds have been synthesized for evaluation of Mo-site or Fe-site complexation hypotheses (Ryle et al., 2000; Paul et al., 2003; Lee et al., 2016). Our focus here is mainly on electron and proton transfer during the process of nitrogenase-catalyzed substrates reduction.

H_2_ production is obligately coupled with N_2_ fixation. N_2_ replaces H_2_ when it binds to reduced FeMoco, and the N_2_/H_2_ molar ratio is 1:1. In fact, H_2_ production is correlated with N_2_ partial pressure, and always exceeds N_2_ fixation amount (Simpson and Burris, 1984). High H_2_ amount and H_2_ burst phenomenon indicate the existence of two electron or proton transfer pathways between P-cluster and FeMoco: one to Fe-site and one to Mo-site (Liang and Burris, 1988). Fe-site accepts electrons to reduce N_2_, with associated H_2_ production. A proposed pathway was confirmed in Gram-negative strains *Klebsiella oxytoca* and *Azotobacter vinelandii*, respectively (Zhao and Li, 2004; Guan et al., 2007). Mo-site accepts electrons to reduce protons to H_2_. α-Ile^423^ may act as a gate for electron transfer to Mo-site, as proposed in our previous study (Guo et al., 2014).

Here, we extend the 2014 study by investigating the function of α-Lys^424^, the residue adjacent to α-Ile^423^. On the basis of bioinformatics analysis and protein structure simulation, we used a single amino acid substitution strategy to replace α-Lys^424^ residue in *K. oxytoca* M5al with arginine (Arg; R), glutamine (Gln; Q), glutamic (Glu; E), and alanine (Ala; A) separately. Comparison at cytological and enzymatic levels of characteristics of wild-type (WT) and mutant strains clarified the role of α-Lys^424^ in substrates reduction of nitrogenase.

## Materials and Methods

### Bioinformatics Analysis

MoFe protein crystal structure data were downloaded from RCSB Protein Data Bank (PDB; ID: 1QGU). Amino acid structure of α-Lys^424^ was analyzed and simulated by PyMOL 2.0.0 software program.

### Construction of Mutants

**FIGURE 1:**
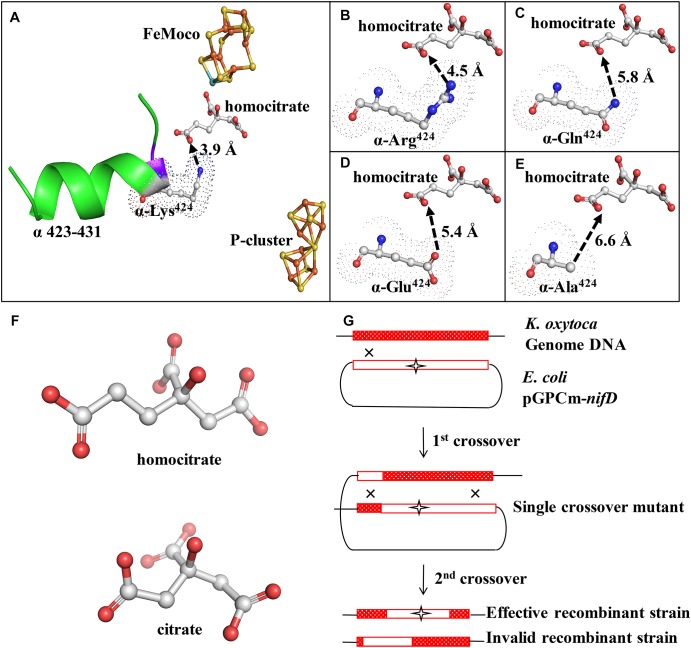
Structure and location of α-Lys^424^ and mutant construction scheme. **(A)** α-Lys^424^ (indicated by dots) is connected directly to α-Ile^423^, and they are located in α-helix (α423-432) (indicated by green ribbon). Distance between N of α-Ile^423^ and O4 of homocitrate is 2.8 Å. Distance between NZ of α-Lys^424^ and O3 of homocitrate is 3.9 Å. **(B–E)** Interactions of substituted amino acids with homocitrate. Gray: C; blue: N; red: O; orange: Fe; yellow: S; cyan: Mo. Simulation and analysis performed using PyMOL software program; PDB ID: 1QGU. **(F)** Structure of homocitrate and citrate. **(G)** Construction of recombinant strains (schematic). *K. oxytoca* received suicide vector pGPCm from *E. coli* and recombined the mutational fragment to homologous sequence of its genome through two-step gene crossover.

Strains and plasmids used in this study are listed in Table 1. For construction of mutants, target *nifD* gene fragment was cloned into the replication vector, pUC18, and the target bases were mutated by PCR. Suicide vector pGPCm containing mutational fragment was constructed and transferred into *K. oxytoca* M5al by bacterial conjugation and fused into *K. oxytoca* M5al genome by recombination. DNA sequencing was used to confirm the target mutation and avoid polar effect. Detailed methods were described in our previous work (Guo et al., 2014). Construction process of mutant strain is also shown in Figure 1G. Primers used for mutagenesis are listed in Supplementary Table S1.

**Table 1:** Plasmids and strains used in the present work.

Plasmids or strains	Characteristics	Reference or source
**Plasmids**		
pUC18	Cloning vector, Amp^r^	Lab collection
pGPCm	Suicide vector for *K. oxytoca*, Cm^r^	Lab collection
pKP1	6.1 kb *nifHDK Eco*RI fragment was cloned into pUC182, Amp^r^	Lab collection
pSD1.4	Vector used for site-directed mutagenesis, Amp^r^	This study
pSDM424R	pSD1.4 derivative containing mutated *nifD* (*nifD*-Kα424R), Amp^r^	This study
pSDM424Q	pSD1.4 derivative containing mutated *nifD* (*nifD*-Kα424Q), Amp^r^	This study
pSDM424E	pSD1.4 derivative containing mutated *nifD* (*nifD*-Kα424E), Amp^r^	This study
pSDM424A	pSD1.4 derivative containing mutated *nifD* (*nifD*-Kα424A), Amp^r^	This study
pGPCm424R	pGPCm with 1.4 kb mutated *nifD* (*nifD*-Kα424R)/*Kpn*I fragment, Cm^r^	This study
pGPCm424Q	pGPCm with 1.4 kb mutated *nifD* (*nifD*-Kα424Q)/*Kpn*I fragment, Cm^r^	This study
pGPCm424E	pGPCm with 1.4 kb mutated *nifD* (*nifD*-Kα424E)/*Kpn*I fragment, Cm^r^	This study
pGPCm424A	pGPCm with 1.4 kb mutated *nifD* (*nifD*-Kα424A)/*Kpn*I fragment, Cm^r^	This study
**Strains**		
*E. coli*		
SM10	*supE44 hsdR th-1 ileuB6 lacY1 tonA21 λpir*, Km^r^	Lab collection
DH5α	*endA1 hsdR17 [r-m+] supE44 thi-1 recA1 gyrA [NalR] relA1Δ[lacZYA-argF] U169 deoR [Ø80Δ{lacZ} M15]*	Lab collection
*K. oxytoca*		
M5al	WT (α-Lys^424^, 424K), Amp^r^	Dr. Burris RH
UNF837	M5al derived strain, *nifV*::*Kan*, Amp^r^Km^r^	Dr. Jufu Huang
424R	*nifD*-Kα424R mutant, Amp^r^	This study
424Q	*nifD*-Kα424Q mutant, Amp^r^	This study
424E	*nifD*-Kα424E mutant, Amp^r^	This study
424A	*nifD*-Kα424A mutant, Amp^r^	This study

*Escherichia coli* strains were grown by standard methods with LB. *K. oxytoca* and its mutants were grown in nitrogen-containing or nitrogen-free medium (Turner et al., 1980). Supplemental antibiotics and their concentrations used for growth of *E. coli* and *K. oxytoca* were: ampicillin (Amp; 100 μg/mL), kanamycin (Km; 50 μg/mL), chloramphenicol (Cm; 15 μg/mL).

### Cell Density (OD_600_), Acetylene, and Proton Reduction Activity Assays

*Klebsiella oxytoca* M5al and its mutant strains were activated on plates and grown overnight in nitrogenous liquid medium at 30°C. Cells were collected, centrifuged, and re-suspended in 60 mL nitrogen-free medium under N_2_ atmosphere, with initial OD_600_ ∼0.25. Every 4 h, OD_600_, acetylene and proton reduction activity were detected. OD_600_ was measured by a UV–visible spectrophotometer (UVmini-1240; Shimadzu, Japan), acetylene and proton reduction activity was measured in a10-mL serum with a sealed stopper according to a previous report (Guo et al., 2014). Ethylene (C_2_H_4_) and H_2_ were, respectively, analyzed by gas chromatography with flame ionization detector (GC-FID) and thermal conductivity detector (GC-TCD) (model GC522, Wufeng Scientific Instruments; Shanghai, China).

### ^15^N_2_ Incorporation Assay

Cells were cultured in nitrogenous medium for 12 h, centrifuged, washed, and suspended in 120 mL nitrogen-free medium. OD_600_ was adjusted to 0.4, and serum bottles were filled with Ar, then 10 mL Ar was removed, and 10 mL ^15^N_2_ gas was injected to induce nitrogenase expression and ^15^N_2_ fixation (negative control: no ^15^N_2_ injection). Cells were incubated at 30°C in water bath for12 h, and bacterial sludge was harvested and freeze-dried. ^15^N_2_ content (mg/g) was measured by Shenzhen Huake Isotope Testing Technology Co., Ltd (Shenzhen, China).

### Detection of *nif* Genes Transcription and MoFe Protein Expression

Samples were collected at three points during diazotrophic growth: the original (5 h), exponential (9 h), and maximal (13 h) activity points. Total RNA was extracted and revers-transcribed. Quantitative real-time PCR (qPCR) was performed using Light Cycler 480 with SYBR Green I Master kit (Roche; Mannheim, Germany). Positive control gene was *groES* (coding chaperone), and target genes were *nifA* (coding positive regulator), *nifL* (coding negative regulator), and *nifD* (coding α-subunit of MoFe protein). Primers used for qPCR are shown in Supplementary Table S1. Relative transcription of mRNA was quantified by 2^−ΔΔCt^ method, as reported by Livak and Schmittgen (2001).

Samples at exponential phase were suspended in lysis buffer and disrupted by ultrasonic. Supernatants were adjusted to equal total protein content, and proteins were separated by Tricine-SDS-PAGE. Antibodies against MoFe protein of *K. oxytoca* M5al were used as probes for Western blotting. Integrated optical density (IOD) of MoFe protein was measured using Image-Pro Plus 6.0 software program (Media Cybernetics; Rockville, MD, United States).

### Purification of MoFe and Fe Proteins From Each Strain

WT or mutant strains were cultured, respectively, in a 42-L autofermenter (Biostat; B. Braun Biotech International GmbH; Gottingen, Germany). When nitrogen source for the first phase was depleted, mutant strains should be added with Arg⋅HCl (1 g/L) to induce nitrogenase expression (Li and Burris, 1983). Acetylene reduction activity was measured every 4 h. Cells were harvested when maximal activity was attained, cells were crushed by ultrasonic under anaerobic condition, and supernatant were collected by high speed centrifugation. MoFe and Fe protein were purified by DEAE-cellulose anion exchange chromatography and preparative gel electrophoresis in order which referred to Li and Burris (1983). The Tris-HCl buffer (pH 7.4) used for protein purification contained 1 mM sodium dithionite to eliminate small amounts of O_2_ in bubbled solution. A major improvement over the previously described method was that 50% sucrose of separation gel was removed to reduce mutational MoFe protein diffusion in separation gel. Purified protein was measured by Tricine-SDS-PAGE and stained by Coomassie Blue, and the protein purity was assessed using BandScan 5.0 software program (Glyko; Novato, CA, United States).

### Calibration of Ratio of MoFe and Fe Protein *in vitro*

Molar ratio of Fe protein to MoFe protein was affected by the flow solution *in vitro*, and it was probably > 2:1 (Kim et al., 1993). It was therefore important to determine this ratio prior to assessing nitrogenase activity. The reaction system contained 5 mM MgATP, 10 mM MgCl_2_, 40 mM MOPS-KOH (pH 7.4), 20 mM sodium dithionite, 40 mM creatine phosphate, and 20 U creatine phosphokinase. MoFe protein quantity was fixed as 20 μg and Fe protein content was increased gradually. The reaction was initiated by addition of 0.9 mL C_2_H_2_, and the C_2_H_4_ production was measured by GC-FID after mixing in a water bath for 30 min at 30°C.

### Activity of Nitrogenase on Catalytic Acetylene and Proton Reduction

The buffer used for nitrogenase activity detection was the same of that used in the calibration assay above. Acetylene reduction activity of nitrogenase was assessed under 90% Ar/10% C_2_H_2_ atmosphere with the appropriate ratio of MoFe and Fe protein that determined by the previous protein ratio calibration. The C_2_H_4_ production was measured by GC-FID. Proton reduction activity was assessed under 100% Ar atmosphere and measured by GC-TCD.

### Kinetics Parameters of Acetylene Reduction

Partial pressure of C_2_H_2_ was set to a series (0, 250, 500, 1000, 2000, 4000, 8000, 12,000, and 16,000 Pa) on the basis of preliminary experiments. C_2_H_2_ reduction activity was detected, and kinetic parameters were calculated according to Lineweaver-Burk plot (Gooch, 2011).

### Detection of Secondary Structure of MoFe Protein

Characteristic three-dimensional structures of proteins (e.g., α-helix, β-sheet, and β-turn) are asymmetric, resulting in an unequal propagation speeds of L and R polarized light beams, and consequent circular dichroism (CD). α-Lys^424^ residue is located in an α-helix of MoFe protein. MoFe protein was diluted to 1 mg/mL with degassing ddH_2_O instead of sodium dithionite, because dithionite had a strong absorption in the UV region; 40 μL protein solution was placed in a 0.1-cm sample cup, and the secondary structure was evaluated by CD spectroscopy with swept wavelength range 185–260 nm (model Chirascan-Plus, Applied Photophysics; Surrey, United Kingdom). The data were analyzed using software program CDNN 2.1 (Micro Focus; Austin, TX, United States).

### Determination of FeMoco Content in MoFe Protein

Presence of unpaired electrons in molecular or atomic orbitals causes compounds to be paramagnetic. Mo^4+^, 6Fe^2+^, and Fe^3+^ of FeMoco give rise to paramagnetic signal. Electron paramagnetic resonance (EPR) signal intensity reflects the FeMoco content when protein concentrations are the same. MoFe protein was diluted to 3 mg/mL and reduced by sufficient sodium dithionite, degassed with high purity dinitrogen, and quantity 150 μL was placed in EPR tubes in the anaerobic glove box (M. Braun; Shanghai, China). Sample tubes were refrigerated sequentially in ice/water mixture (0°C) for 30 s, in drikold/ethanol mixture (−80°C) for 30 s, and finally in liquid nitrogen (−196°C). Low-temperature continuous-wave EPR spectra were recorded on an EPR spectrometer (model E300, Bruker; Karlsruhe, Germany) equipped with an Oxford-900 liquid helium cryostat (temperature range 4–8 K) (Zhang, 2006).

### Electron Transfer Rate Tested by Stopped-Flow Spectrometer

Electron transfer from Fe to MoFe protein is a necessary condition for nitrogenase reduction substrates. The difference of electron transfer rates between WT and mutant nitrogenase helps to determine whether the mutation site affects electron transfer (Igarashi and Seefeldt, 2004); 80 mM MOPS-KOH buffer (pH 7.4), 40 mM sodium dithionite, 20 mM MgCl_2_, and 10 mM MgATP were placed in one reservoir syringe. Fe and MoFe protein were placed in the other reservoir syringe. When proteins come into contact with the buffer, [4Fe-4S] cluster of Fe protein valence state will change and the absorption value at 430 nm will increase. Absorbance increase was monitored by stopped-flow spectrometer (model π^∗^-180, Applied Photophysics; Surrey, United Kingdom). Experiments were repeated five times and the second to fourth were selected to analyze the results. Observed rate constant, *kobs*, for [4Fe-4S]^1+^→[4Fe-4S]^2+^ (F^1+^→F^2+^) step was carried out by Prism 6.0 (GraphPad Software, La Jolla, CA, United States).

## Results

### Simulation of Amino Acid Residues Structure and Construction of Mutants

Structures of α-Lys^424^ and related elements were simulated by PyMOL software. The distance between NZ (ζ-N atom) of α-Lys^424^ and O3 of homocitrate was evaluated as 3.9 Å (Figure 1A). Mutation of α-Lys^424^ was performed to facilitate complete analysis of its function, by weakening interactions between residues without altering the basic structure of nitrogenase. α-Lys^424^ was replaced by Arg, Gln, Glu, and Ala. Structures of substitution residues were also simulated (Figure 1B–E). The distance between branched N and O3 atom of homocitrate theoretically increased when α-Lys^424^ was replaced by Arg (4.5 Å) or Gln (5.8 Å). Glu and Ala had no N atom on the branched chain; their OE1 (ε-O1 atom) and CB (β-C atom) were, respectively, 5.4 and 6.6 Å away from O3 of homocitrate, respectively.

UNF837 is a homocitrate synthetase gene (*nifV*) deleted mutant which is used as a control strain; citrate is a substitute in culture. Therefore, we also provided the simulated structure of homocitrate and citrate in Figure 1F. They have one methylene (–CH_2_–) in difference.

α-Lys^424^ of MoFe protein was selected to site-directed mutagenesis *in vitro* and homologous recombination *in vivo* based on the selected amino acids (Figure 1G). After strict sequencing, four mutants were obtained successfully and termed as 424R, 424Q, 424E, and 424A, respectively.

### Cytological Properties of Nitrogenase

#### Diazotrophic Growth

**FIGURE 2:**
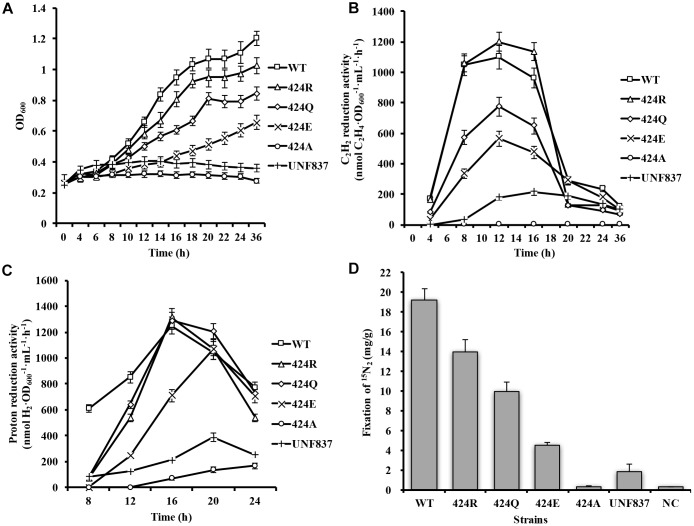
Diazotrophic culture of each strain in shake flask. **(A)** Anaerobic growth in shake flask. Initial OD_600_ of each strain was ∼0.25. OD_600_ was measured every 4 h. **(B)** Acetylene reduction activity during serum bottle culture. 1 mL bacterial solution was placed in 10-mL bottle under 90% Ar/10% C_2_H_2_ atmosphere and reacted for 30 min. **(C)** Proton reduction activity during serum bottle culture under 100% Ar condition. **(D)** Nitrogen fixation activity during serum bottle culture. Cells were collected after 12 h of incubation in ^15^N_2_ atmosphere, and ^15^N_2_ amount was measured by freeze-drying cells.

Diazotrophic growth capability is a fundamental property of nitrogen-fixing bacterial strains. The above-described mutants were cultured in the nitrogen-free medium to evaluate effects of mutations on diazotrophic growth. OD_600_ was measured at 4-h intervals following inoculation. 424A did not grow, while the other three mutants had lower OD_600_ values than that of WT, but a higher value than that of UNF837 in nitrogen-free medium (Figure 2A). After 36 h culture, OD_600_ values of WT, 424R, 424Q, 424E, and UNF837 were, respectively, 1.20, 1.00, 0.85, 0.55, and 0.36. OD_600_ of 424A was about 0.25, similar as the initial value. It seemed that the mutation evidently reduced growth ability.

#### Acetylene Reduction Activity

Acetylene is an alternative substrate for nitrogenase and its product, C_2_H_4_, can be tested easily and quickly by GC-FID. Acetylene reduction activity assay is therefore the most widely used method for estimation of nitrogenase activity. The maximal acetylene reduction activities during cultivation phases of the WT, 424R, 424Q, 424E, and UNF837 were, respectively, 1100, 1200, 774, 564, and 200 (nmol C_2_H_4_⋅OD_600_^−1^⋅mL⋅h^−1^) (Figure 2B). Relative to WT, those of 424Q, 424E, and UNF837 were lower (70, 49, and 18%), and 424A lost acetylene reduction ability. Obviously, 424R was slightly higher (109%), indicating that substitution of Arg did not decrease the catalytic activity of nitrogenase for acetylene.

#### Proton Reduction Activity

Hydrogen emission is a characteristic of nitrogenase, and high hydrogen producing strains have great potential application value. Proton reduction activity of each cell was tested under 100% Ar and measured with GC-TCD. The results are shown in Figure 2C. The maximal proton reduction activity of WT, 424R, 424Q, 424E, 424A, and UNF837 were 1251, 1313, 1288, 1067, 166, and 387 (nmol H_2_⋅OD_600_^−1^⋅mL^−1^⋅h^−1^). 424R and 424Q had similar activity values compared with WT; while 424E, 424A, and UNF837 were lower than that of WT (85, 13, and 31%). 424A lost the ability of diazotrophic growth and acetylene reduction, but retained a very low proton reduction activity, presumably due to the action of hydrogenase in cells.

#### Nitrogen Fixation Activity

Nitrogen fixation activity, a unique characteristic of nitrogenase, directly reflects the nitrogen fixation ability of the nitrogen fixation organisms.^15^N_2_ incorporation assay (see section “Materials and Methods”) for WT was 19.2 mg/g. Corresponding values for 424R, 424Q, 424E, and UNF837 were all lower: 14.0, 10.0, 4.5, and 1.8 (mg/g); respectively, 73, 52, 24, and 13% of WT value (Figure 2D). 424A showed no nitrogen fixation activity; it indicated substitution of Lys by Ala completely eliminated the ability of nitrogenase to reduce nitrogen. These findings were consistent with OD_600_ measurement because nitrogen fixation ability of strains determines growth ability in nitrogen-free medium.

Cytological properties of the four mutants differed from those of WT. Nitrogenase activity was altered by the replacement of a single residue (α-Lys^424^); it seemed that replacement by Arg had the least effect on basic properties of nitrogenase.

### *nifA*, *nifL*, and *nifD* Genes Transcription, and MoFe Protein Expression

The transcription of *nif* genes, and MoFe protein expression were evaluated as possibilities as to causing the observed changes of cytological properties. WT and four mutant strains were cultured in the nitrogen-free medium and harvested at 5, 9, and 13 (h) (respectively, representing original, exponential, and peak phase of acetylene reduction activity). Three *nif* genes were selected to measure. The products of *nifA* and *nifL*, respectively, activate and inhibit *nif* gene cluster transcription as a function of ammonium salt concentration in medium. *nifD* encodes α-subunit of MoFe protein, and it is the site of mutations. qPCR analysis showed that, at each of the three phases, all three *nif* genes transcription level in the mutant strains was 0.7–1.5 folds of WT (Supplementary Figures S1A–C). These differences were not considered significant.

MoFe protein expression at the exponential phase (9 h) was measured by Western blotting, and content was analyzed by Image Pro Plus (Supplementary Figure S1D). IOD varied from 70 to 75, indicating that MoFe protein quantity was similar in the various strains when the total protein was fixed. Thus, *nif* gene transcription and MoFe protein expression were not altered by amino acid substitution. However, MoFe protein of 424A possibly changed in structure, as there was evidently a loss of acetylene and nitrogen reduction activity, and ability to grow in nitrogen-free medium. Therefore, 424R, 424Q, and 424E were used in subsequent experiments, and 424A was excluded.

### Purification of MoFe and Fe Proteins

**FIGURE 3:**
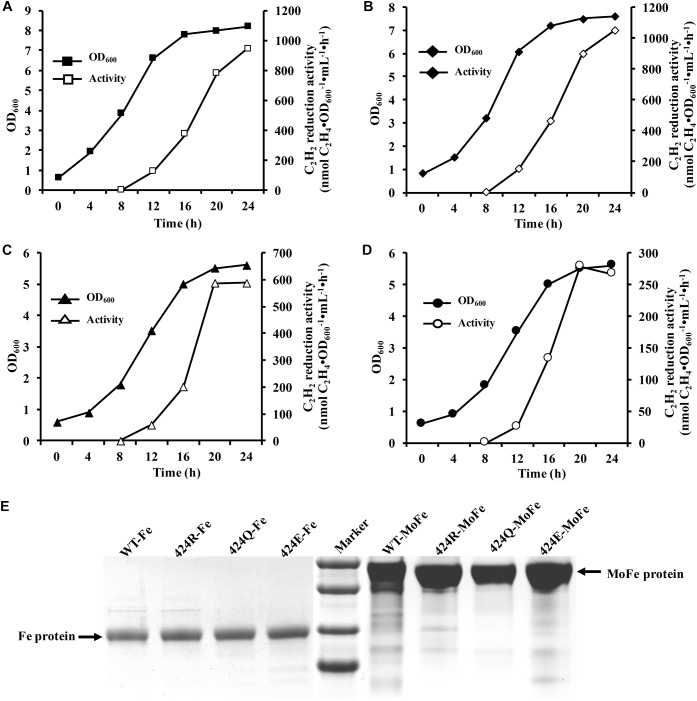
Diazotrophic culture in autofermenter and nitrogenase purification. **(A–D)** Anaerobic growth of WT, 424R, 424Q, and 424E in 42-L autofermenter (30°C, 200 rpm, pH 6.8, automatic regulation by 10 M KOH). **(E)** Tricine-SDS-PAGE analysis of purity of Fe and MoFe proteins.

Selected strains were successfully cultured in 42-L autofermenter by multiple fermentation. Here, one of fermentation batches was shown. Arg⋅HCl (1 g/L) was added to induce nitrogenase expression when the initial NH_4_^+^ was depleted after 8 h culture. Maximal acetylene reduction activity values for WT, 424R, 424Q, and 424E were, respectively, 946, 1051, 588, and 279 (nmol C_2_H_4_⋅OD_600_^−1^⋅mL^−1^⋅h^−1^) (Figure 3A–D), consistently with shake flask results. Sufficient quantities of cells were obtained from each batch of expanded culture. Wet cell weights of collected WT, 424R, 424Q, and 424E by each fermentation were, respectively, 207, 163, 197, and 172 (g), and they were enough to purify the MoFe and Fe proteins.

The crushing of cells, collection of supernatant by centrifugation, and purification of MoFe and Fe proteins were performed under anaerobic conditions as established in our previous studies (Guan et al., 2007). MoFe and Fe proteins of all samples were obtained by anion exchange chromatography and subsequent preparative electrophoresis (Li and Burris, 1983). Wet weights (g) of cells used for nitrogenase purification, and volumes (mL) of obtained MoFe and Fe proteins, are summarized in Supplementary Table S2. Purification efficiency was expressed as mg protein per g wet bacterial sludge. Maximal and minimal efficiencies were, respectively, 0.39 for MoFe protein of 424E and 0.15 for Fe protein of 424Q. The purified proteins were termed as WT-MoFe (or Fe), 424R-MoFe (or Fe), 424E-MoFe (or Fe), and 424Q-MoFe (or Fe). Proteins were stored, respectively, in liquid nitrogen for subsequent experiments.

It was important to ensure nitrogen protection during purification process. Purified proteins were tested by Tricine-SDS-PAGE. As shown in Figure 3E, purities of the Fe and MoFe proteins were > 95% satisfying the requirements for subsequent experiments.

### Enzymatic Properties of Nitrogenase

#### Optimal Molar Ratio of Fe to MoFe Protein for Reaction

The molar ratio of Fe to MoFe protein *in vivo* was 2:1. However, *in vivo* and *in vitro* environments were different and it was necessary to determine the *in vitro* molar ratio prior to analysis of nitrogenase properties. It should be noted that to avoid differences in the reaction process arising from different sources of Fe protein, all reactions were conducted using WT-Fe protein, and MoFe protein quantity was fixed as 20 μg. MoFe protein of the mutant strains and WT showed similar trends; acetylene reduction activity rose gradually as Fe protein content increased, and did not rise further after Fe protein content reached 80 μg. The result is shown in Supplementary Figure S2. Thus, optimal weight ratio of Fe protein to MoFe protein was 4:1. This ratio was used in all subsequent experiments involving the two components.

#### Acetylene Reduction Activity and Kinetic Parameters of Nitrogenase

**FIGURE 4:**
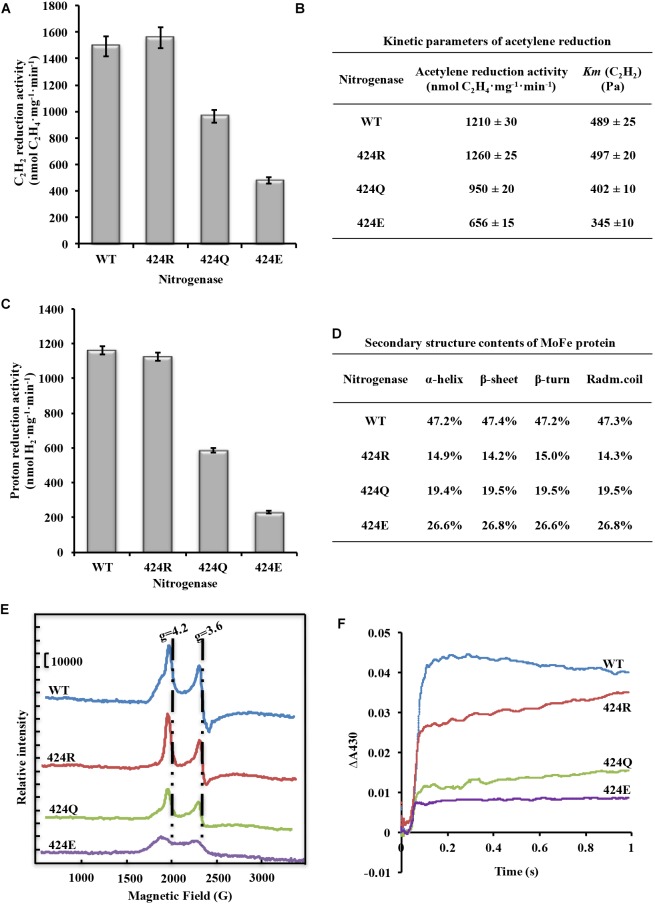
Analysis of nitrogenase properties. **(A)** Acetylene reduction activity of nitrogenase. **(B)** Kinetic parameters of acetylene reduction. **(C)** Proton reduction activity of nitrogenase. **(D)** Secondary structure parameters of WT and altered MoFe proteins. Assay was conducted at room temperature. **(E)** FeMoco content measurement by EPR spectroscopy. Spectrometer settings: power 101 mW; temperature 5 K; microwave frequency 9.38 GHz; amplitude 10 G. **(F)** Electron transfer rate analysis by stopped-flow spectroscopy. Spectrometer settings: wavelength 430 nm, timebase 3 s/10,000 points, bandwidth 0.5 mm; temperature 20°C.

Cytological and enzymatic acetylene reduction activities are two aspects of nitrogenase activity. Acetylene reduction activity of pure nitrogenase, determined under 90% Ar/10% C_2_H_2_, was 1493 nmol C_2_H_4_⋅mg^−1^⋅min^−1^ for WT. Values for 424R, 424Q, and 424E were 1559, 967, and 479 (nmol C_2_H_4_⋅mg^−1^⋅min^−1^), which were 104, 65, and 32% of WT, respectively (Figure 4A). Enzyme kinetics reflects the ability of enzyme binding substrate and catalytic reaction rate. Amino acid mutation near FeMoco is likely to reduce the efficiency of acetylene binding to nitrogenase. Partial pressure of acetylene changed to detect kinetic parameters of acetylene reduction by nitrogenase. The trend of maximum acetylene reduction activity was consistent with the above results, and *Km* of WT, 424R, 424Q, and 424E were, respectively, 489, 497,402, and 345 (Pa) (Figure 4B).

#### Proton Reduction Activity of Nitrogenase

Proton reduction activity is one of fundamental properties of nitrogenase. Usually, H_2_ production accompanies N_2_ reduction. The detection was determined under 100% Ar atmosphere. The results showed that the activity was very similar for WT and 424R: 1162 and 1124 (nmol H_2_⋅mg^−1^⋅min^−1^), respectively (Figure 4C). In contrast, activities of 424Q and 424E were only 587 and 231 (nmol H_2_⋅mg^−1^⋅min^−1^), which were 50 and 20% of WT value.

#### The Secondary Structure of MoFe Protein

α-Lys^424^ is located in the α-helix (α423-431) of MoFe protein. To evaluate effect of mutation on the α-helix, secondary structure of MoFe protein was examined by CD spectroscopy. The results indicated that each of the three substituted MoFe proteins displayed the same characteristic α-helix peaks (194, 208, and 222 nm) as MoFe protein of WT. α-helix and β-sheet contents of all nitrogenases were in the range 47.2–47.4 and 14.3–15.0% (Figure 4D). These results were similar to that obtained previously with crystal structure of nitrogenase, 48% and 15% (Mayer et al., 1999). These indicated that there were no notable differences in contents of the secondary structures. Thus, replacement of α-Lys^424^ did not cause major structural alternation of MoFe protein. MoFe protein is large (∼240 kD) and complex (1994 amino acids), it is not surprising that replacement of a single residue did not greatly alter its integral structure. This is what we expected, that it is meaningful to judge the role of target amino acid according to the result of property analysis.

#### Content of FeMoco in MoFe Protein

Because H_2_ production of nitrogenase is generally correlated with reduction of FeMoco content in MoFe protein (Durrant et al., 2006). We therefore proceeded to investigate FeMoco content of MoFe protein in the mutant strains. EPR spectroscopy is useful for qualitative and quantitative detection of unpaired electrons, which are present in molecular or atomic orbitals of many compounds. The action of an external magnetic field on unpaired electrons results in energy level splitting and consequent generation of an EPR signal. The first differential line of absorption is recorded by an EPR spectrometer.

FeMoco is the active center of nitrogenase, and its Mo^4+^, 6Fe^2+^, and Fe^3+^ are the source of EPR signal. Reduced FeMoco has an S = 3/2 EPR signal, and signal intensity reflects FeMoco content when protein concentration remains constant. Reasons for enzyme activity change were investigated by recording EPR spectra of MoFe proteins at 5 K, 101 mW. Peak locations for four MoFe proteins were the same (Figure 4E). When g factor was 4.2, peak intensity (crest minus baseline) of WT was 49,100, while intensities of 424R, 424Q, and 424E were 49,200, 32,000, and 14,900; they were, respectively, 100, 65, and 30% of WT value. When g factor was 3.6, peak intensity (crest minus trough) of WT was 55,400, while intensities of 424R, 424Q, and 424E were 59,100, 33,400, and 15,200; they were, respectively, 107, 60, and 27% of WT value. Ratios did not differ significantly for g factor 3.6 vs. 4.2. One of the two peaks could therefore be selected for representation of intensity and we chose to base percentages on the ratio of *g* = 4.2 of WT EPR signal intensity. FeMoco content of 424R was identical to that of WT, while those of 424Q and 424E were lower (∼65 and 30% of WT value). Amino acid substitution in MoFe protein did not result in new EPR signal. g factor and line width were also unchanged. Our findings indicated that mutations of α-Lys^424^ did not alter FeMoco structure but did affect insertion of FeMoco in MoFe protein. Reduction of FeMoco content resulted in decreased proton reduction activity.

#### Electron Transfer Between Fe and MoFe Protein

When reduced Fe protein transfers an electron to resting state MoFe protein, the valence state of [4Fe-4S] cluster will go up from +1 to +2 (F^1+^→F^2+^). F^2+^ will be re-reduced to F^1+^ (F^2+^→F^1+^), and this process involves the dissociation of Fe and MoFe protein. As the process of re-reduction is complicated, we only consider the process F^1+^→F^2+^, and its observed rate constant, *kobs*, is calculated.

As shown in Figure 4F, oxidized [4Fe-4S] cluster curves were all fit to a single exponential function. Through curve fitting by exponential functions, it was found that *kobs* of 424R, 424Q, and 424E were 17.0, 17.3, and 16.8 s^−1^, respectively. Their electron transfer rates were almost equal to WT (17.4 s^−1^). But absorbance of WT, 424R, 424Q, and 424E were 0.044, 0.035, 0.023, and 0.015. The depressed absorbance values of mutations demonstrated a decreased amount of electron transfer.

## Discussion and Conclusion

### Amino Acid Residues Around FeMoco Are Immutable

FeMoco is situated deep below MoFe protein and surrounded by amino acid residues, supported by α-subunit mainly. The residues play varying roles in catalytic properties and orientation. α-Lys^424^ is located near homocitrate long arm. Homocitrate serves as a terminus of FeMoco. *nifV*^−^ mutation, in which homocitrate was replaced by citrate resulted in significant decreases of acetylene, proton, and nitrogen reduction activity, indicating an important role of homocitrate in nitrogenase activity (Liang et al., 1990). Nitrogenase undergoes protonation during the catalytic cycle, but the specific protonation sites remain unclear (Hoffman et al., 2014). Fe2 and Fe6 atoms were considered as possible protonation sites and α-Gln^190^ and α-His^194^ residues were experimentally replaced by α-Lys^190^ and α-Gln^194^ to support the view (Zhao and Li, 2004; Guan et al., 2007). α-Lys^190^ substitution affected electron transfer, while α-Gln^194^ substitution impaired nitrogen fixation activity of nitrogenase. The α-alkoxy groups of homocitrate, functioning as bidentate ligands of Mo atom, are also possible protonation sites (Chen et al., 2014; Wang et al., 2018). α-Lys^424^ residue is likely involved in protonation of α-alkoxy groups. Structurally, Arg is similar to Lys, and both of them are basic amino acids. They tend to form water clusters through hydrogen bond, and the water clusters steadily provide protons for α-alkoxy groups. Gln and Glu are structurally different from Lys. Gln has an N atom on the R-group, resulting in formation of water clusters; however, the distance between α-Gln^424^ and homocitrate long arm is larger (5.8 Å), and activity of 424Q is therefore lower than that of 424R. Gln and Glu are generally similar in structure, but the groups on Glu’s R-group have no N atom, and O atom forms a weaker hydrogen bond. The change of nitrogenase activity for 424E is more moderate than that of 424Q.

The four amino acids were also substituted in UNF837 strain. As shown in Supplementary Table S3, although their growth level showed no obvious difference except the UNF837/424A strain, the acetylene reduction characteristics of each double mutant were significantly lower than *nifV*^−^ single mutant. They were not only replaced the homocitrate in MoFe protein, but also disrupted the microenvironment of active sites due to the replacement of amino acids. That was why they lost enzymatic properties more thoroughly.

Our findings supplemented the structure/function relationship in nitrogenase. The amino acids surrounding FeMoco are precisely selected by evolution in nitrogen-fixing microorganisms.

### Reduction of FeMoco Content Decreases H_2_ Release

Cytological and enzymatic H_2_ production is inconsistent, and percentages of cytological production are higher than those of enzymatic, indicating that there are other enzymes in *K. oxytoca*, for example hydrogenase, that can reduce the proton to H_2_. Enzymatic H_2_ production reflected ability of nitrogenase reduction proton. And the process is affected by numerous factors. For example, it is inhibited by N_2_ in WT and by carbon monoxide (CO) when the α-Gln^190^ residue is replaced by Lys (Zhao and Li, 2004). Substitution of α-Pro^423^ for α-Ile^423^ results in 21% decrease of H_2_ production relative to WT (Guo et al., 2014). In this study, H_2_ production was correlated with FeMoco content. The decrease of H_2_ production in 424Q and 424E was attributable to FeMoco content reduction. It indicated that the substitution of these two amino acids does not guarantee the successful insertion and catalytic function of FeMoco.

### α-Lys^424^ and α-Ile^423^ Jointly Coordinate Insertion and Maintain Function of FeMoco

The acid-base property of amino acid at α-424 site obviously affects the content of FeMoco in MoFe protein. Alkaline Lys and Arg do not affect FeMoco content, while neutral Gln or acidic Glu decrease FeMoco content to varying degrees. As Schmid et al. (2002) reported previously, alkaline amino acids contributed to forming a positively charged funnel to assist FeMoco insertion on the basis of MoFe protein crystal structure analysis. Neutral or acidic amino acid substitution was not conducive to the insertion of negatively charged FeMoco; this resulted in the formation of some apo-MoFe proteins. α-Lys^426^ in MoFe protein of *A. vinelandii* corresponds to α-Lys^424^ in MoFe protein of *K. oxytoca*. As far as crystal structure was concerned, α-Lys^424^ was one of the alkaline amino acids formation of the funnel. Our experimental results coincided with previous reports. In addition, Siegbahn Per (2018) reported that α-Lys^424^ could form a direct hydrogen bonding with long arm of homocitrate. Thus, α-Lys^424^ possibly serves to immobilize the FeMoco after insertion. α-Ile^423^ is also located close to homocitrate long arm (2.82 Å), and this short distance results in a strong hydrogen bonding according to Dance (2015) results. Therefore, α-Lys^424^ and α-Ile^423^ jointly maintain the location of homocitrate in MoFe protein.

Electron transfer between the Fe and MoFe proteins is essential for nitrogenase activity (Seefeldt et al., 2012). Amino acid residues surrounding FeMoco contribute to this process. α-Gln^190^ and α-His^194^ are involved in electron transfer from P-cluster to S2B of FeMoco. In this study, acetylene and proton reduction activities of mutants were correlated with the FeMoco content of MoFe proteins, but nitrogen fixation activity of the mutants reduced to a greater degree than acetylene reduction activity, presumably because of increased potential around FeMoco. Results of stopped-flow also provided indirect evidence for FeMoco potential increase, because for 424R the number of electrons FeMoco received decreased more obviously than WT when their electron transfer rate and FeMoco contents were the same. The lower the potential of a metal cluster, the stronger its reducibility. Amino acids interact with metal clusters and affect their potential (Hough et al., 2005). α-Lys^424^ most likely alters the potential of the FeMoco by opening the chelate ring and forming a hydrogen bond with the carboxylate group (–CH_2_CH_2_CO_2_–) of homocitrate (Durrant et al., 2006). Dynamic change of the homocitrate structure may reduce the number of electrons FeMoco received, thus decreasing its reducibility. It is not possible to identify the binding site of N_2_, because regardless of where the binding occurs, high potential of FeMoco will inhibit the nitrogen fixation activity.

Researchers have been attempting for many decades to construct an efficient “nitrogen fixation plant”, but the dream has not yet been achieved. The major obstacle is the low activity observed when nitrogenase is expressed in *E. coli*, yeast, chloroplast, etc. (Ivleva et al., 2016; Allen et al., 2017; Burén et al., 2017; Yang et al., 2017). The low activity is due to unsuitable surrounding environments, such that MoFe protein does not fold correctly, or FeMoco cannot assemble on MoFe protein. The role of amino acid residues around FeMoco is not monotonous and should not be treated indiscriminately in research designs.

In conclusion, the results of the present study supplemented experimental evidence of alkaline α-Lys^424^ to coordinate insertion of FeMoco to MoFe protein, in combination with certain other amino acids; and demonstrated its function in maintaining low potential of the microenvironment to guarantee efficient catalytic activity of nitrogenase.

## Author Contributions

JL, YL, and WJ conceived the project and designed the experiments. LS, PL, QG, WJ, and CZ performed the experiments and data analysis. JL, CZ, and AB provided useful suggestions and discussion. LS and YL wrote the manuscript. All authors read and approved the finalized manuscript.

## Conflict of Interest Statement

The authors declare that the research was conducted in the absence of any commercial or financial relationships that could be construed as a potential conflict of interest.
