# Supplementary material for: α-Lys424 Participates in Insertion of FeMoco to MoFe Protein and Maintains Nitrogenase Activity in Klebsiella oxytoca M5al
Source: Front Microbiol. 2019 Apr 16;10:802. doi: 10.3389/fmicb.2019.00802 (PMC6477116; doi:10.3389/fmicb.2019.00802)
Supplement: Supplementary file 1 [file Data_Sheet_1.PDF]

Supplementary Materials

Supplementary Figures

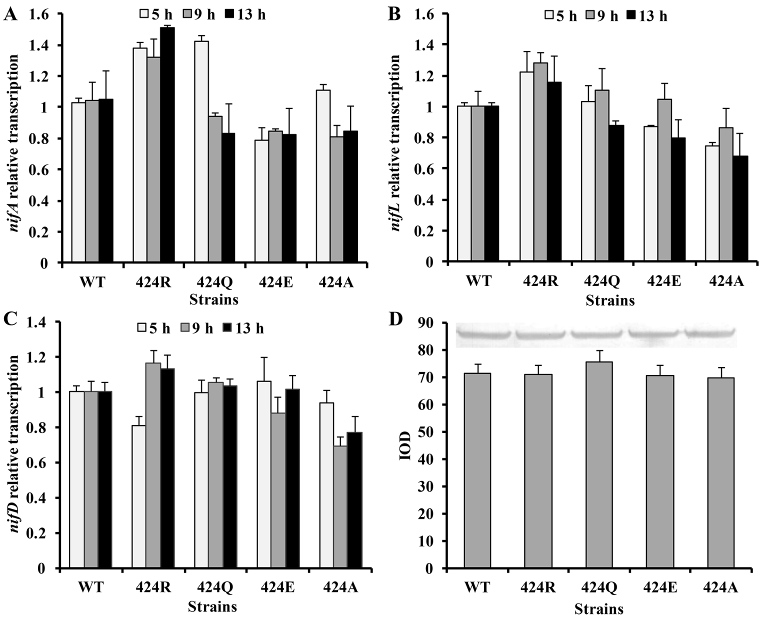

Figures S1 Transcription of selected *nif* genes, and MoFe protein expression. **(A)-(C)** Relative transcription of *nifA*, *nifL*, and *nifD*. Cells were cultured in 250-mL serum bottle and harvested at 5, 9 and 13 h. Harvest time was set on the basis of acetylene reduction activity, and transcription was measured by qPCR. **(D)** Western blotting of MoFe protein, and IOD analysis. Total protein amount was the same (30 μg) for each lane. Blots are shown at top.

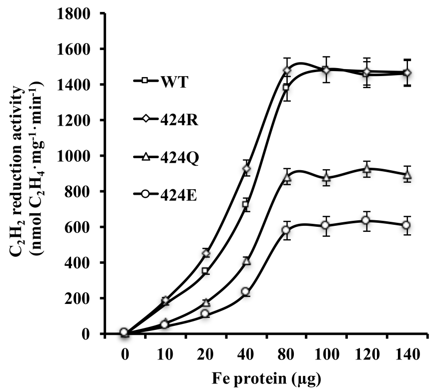

Figures S2 Calibration the acetylene reduction activity of nitrogenase. The reaction system contained 10 mM $MgCl_2$, 5 mM MgATP, 20 mM dithionite, 40 mM MOPS-KOH (pH 7.4), 40 mM creatine phosphate, and 20 U creatine phosphokinase. MoFe protein was 20 μg, and Fe protein was increased gradually to 140 μg. The optimal mass ratio of Fe and MoFe protein was 4:1.

Supplementary Tables

Table S1 Primers used for mutagenesis and qPCR in this study.

Primers	DNA sequences (5'-3')
424R-F	CCGGCATCCGCGAAAAATATATC
424R-R	AGATATATTTTTCGCGGATGCCGG
424Q-F	CCGGCATCCAGGAAAAATATATC
424Q-R	AGATATATTTTTCCTGGATGCCGG
424E-F	CCGGCATCGAGGAAAAATATATC
424E-R	AGATATATTTTTCCTCGATGCCGG
424A-F	CCGGCATCGCCGAAAAATATATC
424A-R	AGATATATTTTTCGGCGATGCCGG
groES-F	ATCATCGCTGTCGGTAAGG
groES-R	TGCCAGAATGTCGCTTTC
nifA-F	TGATGATTCTGCCGACCTC
nifA-R	GGAGACCTGACGGATAATGTC
nifL-F	TCTACCAGCAGATGTGGC
nifL-R	CTCCAGCTCGCCCTGGGGT
nifD-F	CATCGGCGACTACAACA
nifD-R	TCCATCCACGGAATCTGA

Note: target mutational bases are underlined in the primer sequence.

Table S2 Purification of Fe protein and MoFe protein.

Strains	Cell wet weight (g)	Protein component	Volume (mL)	Concentration (mg/mL)	Purification efficiency (mg/g)
WT	120	Fe	9.60	4.22	0.34
		MoFe	4.70	9.05	0.36
424R	90	Fe	3.50	4.34	0.17
		MoFe	5.00	3.15	0.18
424Q	90	Fe	2.60	5.25	0.15
		MoFe	4.00	6.04	0.27
424E	120	Fe	5.30	8.09	0.36
		MoFe	6.50	7.12	0.39

Table S3 Cell growth and acetylene reduction activity of UNF837 and its mutants

Strains	OD_{600}	Acetylene reduction activity (nmol $C_2H_4 \cdot OD_{600}^{-1} \cdot mL \cdot h^{-1}$)
UNF837	0.359±0.05	215.04±10.91
UNF837/424R	0.329±0.04	180.32±11.33
UNF837/424Q	0.335±0.05	108.19±7.08
UNF837/424E	0.341±0.05	64.55±4.25
UNF837/424A	0	0
